# Analytical Modelling of MSW Landfill Surface Displacement Based on GNSS Monitoring

**DOI:** 10.3390/s20215998

**Published:** 2020-10-22

**Authors:** Dana Adamcová, Stanislav Bartoň, Piotr Osinski, Grzegorz Pasternak, Anna Podlasek, Magdalena Daria Vaverková, Eugeniusz Koda

**Affiliations:** 1 Faculty of AgriSciences, Mendel University in Brno, Zemědělská 1, 613 00 Brno, Czech Republic; dana.adamcova@mendelu.cz (D.A.); magda.vaverkova@uake.cz (M.D.V.); 2 Faculty of Electrical Engineering Automatic Control and Informatics, Opole University of Technology, Prószkowska 76, 45-758 Opole, Poland; s.barton@po.edu.pl; 3 Institute of Civil Engineering, Warsaw University of Life Sciences—SGGW, Nowoursynowska 159, 02-776 Warsaw, Poland; piotr_osinski@sggw.edu.pl (P.O.); grzegorz_pasternak@sggw.edu.pl (G.P.); anna_podlasek@sggw.edu.pl (A.P.)

**Keywords:** waste, landfill displacements, landfill settlement model, geodetic survey

## Abstract

Displacements of landfills play an important role in the reclamation process and geotechnical safety improvement of such sites. Landfill settlements are defined as a vertical displacement of waste body due to compression, degradable nature of the waste, and creep phenomenon of the waste particles. Waste composition is more diverse than natural soil. Thus, it has to be properly placed and compacted since the landfill body will continuously settle down. Several models of the landfill displacement estimation have already been developed. The aim of the present study was: (i) to review the methods of landfill settlements computation and (ii) to propose the model allowing landfill body displacements simulation based on monitoring datasets applying a Global Navigation Satellite Systems (GNSS) measurement. The new model employs Gauss-Newton iteration and Runge-Kutta methods to estimate landfill surface displacements. The objectives were to analyse and mathematically describe the landfill body displacements. The GNSS geodetic survey and computations allowed concluding that the landfill body has been transformed over the years. The results revealed that the curves of waste displacement are in agreement with the measured total displacement of the landfill, and all curves corresponding to waste displacement are perpendicular to the active edge of the landfill. In the period of a maximum of 4.5 years after the waste deposition with a layer of up to 16.2 m thickness, the phenomenon of expansion was observed, which then disappears, and more settlement occurs due to the gravity of upper layers. The analysed landfill as a whole does not experience significant displacements. Neither of the slope failures are observed, even for large inclination.

## 1. Introduction

Landfills represent a method appropriate and acceptable for the municipal solid waste (MSW) disposal. However, proper solid waste management (WM) is crucial for protecting both human health and a natural environment [1,2]. Waste disposal in landfills puts up short-term and long-term challenges to research due to mutual interactions of hydraulic, biodegradation, and mechanical phenomena in their environment. McDougall [3] reported that, during the short-term observations, the presence of organic substances in the waste leads to the high compressibility of the landfill body. In the long-term scenarios, the main issue appears as a loss of waste matter caused by biodegradation of organic waste (OW) compounds of which the mechanical consequences have not been fully investigated yet [4]. Landfill settlements are generally defined as a vertical displacement of the waste body due to MSW compression, rearrangement of the particles, and biological degradation of the MSW organic matter (OM) [5].

Landfill settlement measurements determine the geotechnical safety of such a structure and are crucial for the future development plan of restored landfill. Displacement of MSW is a complex process that depends on mechanical factors due to waste overburden and on physical, biological, and chemical processes occurring on such structures [6,7,8,9]. The factors affecting displacement process the most are morphological composition, thickness of waste layers, stress history depending on waste compaction method, the initial density of waste material due to compaction, waste particles’ size distribution, degradable components content, leachate level, drainage characteristics, atmospheric conditions (moisture content, temperature), and time that the waste has been disposed on the landfill [10,11,12]. Top et al. [13] indicated that the settlement of MSW can be enhanced by the aeration and leachate recirculation.

MSW material is highly compressible, which may induce considerable compression [14]. Prior to MSW disposal in the landfill, the waste has to be properly placed and compacted since waste dumps as well as the whole landfill body will settle down. During the landfill body compaction and engineering, which take place immediately after the waste placement, it is assumed that MSW affects itself by its own weight, or, additionally, another load that is co-acting, e.g., caused by the compactor. In the literature, this process is defined as the initial levelling of the landfill body, which can be of either a mechanical or a structural character [15].

In MSW landfills, there are also other influences that have to be taken into account and utilized for the future development of landfill body behaviour such as leachate production or development of landfill gas [16,17]. Landfill body settlement is a physical phenomenon caused by the existence of hollow spaces (voids) in the deposited waste. Waste layers tend to fill these empty spaces, which leads to waste compression and, subsequently, to increased density in the landfill body. Settlement of landfill bodies represents an important issue to be faced during handling MSW in the landfill as well as during the landfill operation itself. Landfills settle due to the process of waste handling and also due to chemical and microbial activities. These processes are affected by time and a range of other factors such as composition and pH of leachates or their temperature.

Settlement of landfill bodies is a long-term and complex process that has to be monitored because waste stabilization and settlement of landfill body play an important role in the reclamation of the landfill and aftercare following its closure. During the process of waste filling up the landfill for reclamation purposes, landfill managers strive for the maximum utilization of the landfill body space, i.e., for the minimization of airspaces in wastes.

In the past, several models have been developed with the goal of estimating waste stabilization in the landfill body and landfill settlements [18,19,20,21,22]. El-Fadel and Khoury [23] presented a list of models developed before 2000 and classified the models into four categories: models based on soil mechanics, rheological models, empirical models, and models based on biodegradation processes. A majority of current models of waste stabilization in the landfill body dwell on one or even more of those models. El-Fadel and Khoury [23] identified pros and cons of simple models as compared with more complex ones and claimed that the models should be based on more detailed field data. According to studies performed by Van Geel and Murray [24], it can be concluded that more field data are required to verify different settlement models and their respective parameters, especially because the selection of parameters inadequate for considered conditions leads to the overestimation of the real settlement values.

Recent studies from Brazil [5] showed that settlement behaviour of MSW due to internal and external environmental factors can be simulated by an experiment performed in a lysimeter. Moreover, cited authors revealed that the interpretation of factors determining the range of landfill settlements can be enhanced with the application of statistical tools such as descriptive analysis and principal component analysis. The research conducted by Araújo Neto et al. [25] also confirmed that the settlements that occur in a landfill over time can be assessed using a simple linear regression model based on a lysimeter experiment. The similar study was performed by Bareither and Kwak [26] who monitored the range of settlements at various depths of the designed waste column. Moreover, the use of the unmanned aerial vehicles (UAV) for the assessment of landfill settlements is the subject of scientific studies. In this regard, Baiocchi et al. [27] presented the approach aiming at the analysis of optical images in order to monitor the morphological variations occurring in the landfill. Remez et al. [28] also highlighted that every simulation of the landfill settlement should be performed with regard to the characteristics of subsoil. The authors established that, with the same conditions of a studied area, the parameters of the underlying soil layer significantly affect the settlement range, which means that smaller settlements occur for clays than for sands.

In connection to the issues presented above, the aim of this study was: (i) to review the methods applied in landfill settlements calculations and (ii) to propose the model aiming at the prediction of landfill body displacements, based on actual measured data. The model is based on [*x*, *y*, *z*] of 350 points forming the surface of the landfill body, 40 points on the circumference of the landfill body, which, together with the other six points, define the shape of the subsoil and [*x*(*t*), *y*(*t*), *z*(*t*)] of 15 points that define the displacement of the surface of a landfill body over time. Any conclusion on landslides and the like is not the subject of this study, even though they can be coupled together with large deformations in the landfill slopes.

## 2. Materials and Methods

### 2.1. Models of the Landfill Settlement Prediction—A Review

Displacement assessment is a challenging engineering task. In fact, due to non-homogeneous composition of waste, applying the mean values of parameters used for computations is unjustified. Each MSW landfill needs to be analysed individually. It is recommended to use several sophisticated computational models for a single case study to fully verify and validate the predicted values [29]. Displacements of waste, just like in natural soil, decrease with time. For the purpose of MSW landfill displacement computations, there have been a number of sophisticated models developed by different researchers [6,30]. Nevertheless, Simões and Catapreta [8] suggested that long-term settlement prediction in the MSW landfill may not be restricted to the use of a single model, but the use and comparison of different models should be applied to define final settlements, mainly during the early stages of planning and designing the landfill. Gao et al. [14] pointed out that, generally, there are two methods aiming at the determination of the MSW landfill settlements. The first one is based on the application of the Finite Element Methods in accordance with the constitutive model, whereas the second one is the compression model related to the layer-wise summation. Nevertheless, all of the developed and proposed models require the set of appropriate parameters [26]. The assessment of proper model parameters is typically conducted based on field-scale data, laboratory experiments, or empirical relationships with waste characteristics [31]. Ivanova et al. [32] highlighted that the long-term scale laboratory experiments are unique for identifying the key mechanisms that contribute to waste settlement under various load conditions. The landfill deformation process should be monitored and forecasted. The rate of decomposition of organic substances in the landfill and the associated deformations also depends on the temperature in the waste deposit [33].

There are also software programs developed by the integration of the space and time discretization to assess the settlement algorithms. Tahmoorian and Khabbaz [34] predicted time-dependent settlements considering related parameters (e.g., leachate and gas generation, moisture content, and compression coefficient) with the use of the MATLAB software. Cuartas et al. [35] proposed that settlements can be calculated according to the morphology, moisture content, and decomposition in selected time steps. In practice, most commonly used models involve the analysis of two or three displacement stages.

When compared to soil, waste material exposes much higher compressibility, which determines settlement of the entire landfill. The main mechanisms of the displacement process that could be observed in a long time-scale monitoring are primary settlements (as an effect of waste mass and compaction process), decomposition settlements (due to biological and chemical processes), and residual settlements (as a result of creeping). The assessment of settlements is crucial to evaluate the volume of the landfill body, but also to properly design drainage systems, degassing systems, capping, and linear on the landfill [14]. Major differences in the settlement could also cause a local instability of the landfill slopes. For the purpose of much more accurate settlement predictions, the compressibility parameters of waste need to be first investigated. Once such parameters are established, more sophisticated models could be applied.

Ling et al. [36] proposed hyperbolic correlation to assess the range of landfill displacements (Equation (1)).

(1) $$S=\frac{t}{\frac{1}{v_{0}}+\frac{t}{S_{ult}}}$$

where *S*—displacement difference at time *t*, *t*—calculated and initial time difference (*t = t_i_ − t*_0_), *ν*_0_—initial velocity in time *t*_0_, *S_ult_*—ultimate displacement at time *t*–*∞*.

It must be highlighted that several methods developed for the assessment of the landfill settlement are based on the premises of soil mechanics. Sowers [6] applied the model of consolidation and revealed that the long-term compression of wastes is related to creeping and the biodegradation and can be expressed with the use of the secondary compression index *C_α_* (Equation (2)).

(2) $$\Delta H=HC_{c}{}^{*}\log\frac{P_{0}+dP}{P_{0}}+HC_{\alpha }\log\frac{t_{2}}{t_{1}}$$

where Δ*H*—the settlement value due to primary and secondary consolidation, *H*—initial thickness of waste layer, C_C_*—primary compression ratio, *P*_0_—existing overburden pressure in the middle of the layer, *dP*—increment of overburden pressure acting in the middle of the layer resulting from the addition of the subsequent layer, *C_α_*—secondary compression index, *t*_2_—ending time for initial compression, *t*_1_—ending time for long-term settlement.

Although the Sowers model is the most commonly used in practical aspects, Hadinata et al. [37] made an attempt to modify the Sowers (1975) formula by adding a correction factor representing the initial settlement occurring prior to the addition of waste layers. Proposed modification resulted in a decrease in the difference between estimated and actual settlement values, from 17–24% to below 5%. Xu et al. [38] indicated that the Sowers model is more suitable for predicting settlements at landfills with low food waste content than at landfills characterized by a high content of food wastes.

Bjarngard and Edgers [39] assumed that total settlements of a landfill are the sum of primary settlements (*S_C_*), and settlements are derived from mid-secondary compression (*S_w_*_1_) and mechanical compression of waste (*S_w_*_2_). The formula is shown below (Equation (3)).

(3) $$S=S_{c}+S_{w1}+S_{w2}=H\cdot C_{C\varepsilon }\cdot \log\frac{\sigma_{0}+\Delta \sigma }{\sigma_{0}}+H\cdot C_{\alpha \varepsilon 1}\cdot \log\frac{t_{2}}{t_{1}}+H\cdot C_{\alpha \varepsilon 2}\cdot \log\frac{t_{3}}{t_{2}}$$

where *C_Cε_*—initial compressibility factor, *C_αε_*_1_—secondary compressibility factor, *C_αε_*_2_—secondary mechanical compressibility factor, *σ*_0_—mean stress of waste layer, Δ*σ*—stress increase in the waste layer, *t*_1_—time of initial compressibility, *t*_2_—time of secondary mechanical compressibility, *t*_3_—prediction time.

Hossain and Gabr [40] developed the model allowing for the long-term settlement prediction with respect to the biodegradation phenomena (Equation (4)).

(4) $$\frac{\Delta H}{H}=C_{\alpha i}\log\frac{t_{2}}{t_{1}}+C_{\beta }\log\frac{t_{3}}{t_{2}}+C_{\alpha f}\log\frac{t_{4}}{t_{3}}$$

where *C_α_*_1_—compression index, *t*_1_—time required for the ending of the initial compression, *t*_2_—time required for the evaluation of compression, *C_β_*—biodegradation index, *t*_3_—time required for the ending of biological compression, *C_αf_*—creep index, *t*_4_—time required for the creep at the end of biodegradation.

Several researchers proposed empirical models to assess the range of settlements in MSW, used quite commonly in geotechnical engineering. Gibson and Lo [41] developed the model to predict the long-term total settlements by proposing the following formula (Equation (5)).

(5) $$\frac{\Delta H}{H}=\Delta \sigma a+\Delta \sigma b(1-e^{-\left(\frac{\lambda }{b}\right)t})$$

where Δ*σ*—compressive stress, *a*—primary compressibility parameter, *b*—secondary compressibility parameter, λ/b—rate of secondary compression, *t*—time since load application.

Yen and Scanlon [42] indicated that landfill settlement can be described using the logarithmic function (Equation (6)).

(6) $$\Delta H=H\left[\alpha +\beta \;\log\left(t-\frac{t_{c}}{2}\right)\right]$$

where *α*, *β*—fitting parameters, time since the beginning of filling, *t_C_*—construction period.

Based on nine years displacements velocity and observation of 30-m high landfills, mentioned authors proposed a logarithmic formula for calculating landfill displacement velocity (Equation (7)).

(7) $$v=\frac{dS}{dt}=m-n\log t$$

where: *ν*—displacement velocity, *dS*—displacement increase, *t*—time elapsed from the end of landfilling, and *m* and *n*—constant values.

Values *m* and *n* in the previously mentioned formula can be set according to the following expressions (Equations (8) and (9)).

(8) $$m=0.00095\;H_{f}+0.0323$$

(9) $$n=0.00035\;H_{f}+0.0167$$

where *H_f_*—height (thickness) of the landfill in feet.

Edil et al. [43] presented the formula describing the time-dependent deformation under constant stress (Equation (10)).

(10) $$\Delta H=H\;\Delta \sigma M^{\prime }{\left(\frac{t}{t_{r}}\right)}^{N^{\prime }}$$

where *M*′—reference compressibility, *N*′—compression rate, *t*—time since load application, *t_r_*—reference time.

Edil et al. [43] also demonstrated an exponential model for calculating displacement velocity *v* at individual time steps in accordance with the formula (Equation (11)).

(11) $$v=\frac{dS}{dt}={\left(\frac{a}{t}\right)}^{b}$$

where: *a*, *b*—positive empirical values.

Ling et al. [36] proposed a hyperbolic function to calculate landfill settlement at a given time when the final settlement is known, following the formula below (Equation (12)).

(12) $$\Delta H=\frac{t}{\frac{1}{\rho_{0}}+\frac{t}{S_{f}}}$$

where: *ρ*_0_—initial rate of settlement, *t*—time since load application, *S_f_*—final settlement.

Marques et al. [21] performed the composite biological model to predict landfill deformation, analytically expressed as Equation (13):

(13) $$\varepsilon =\frac{\Delta H}{H}=C_{c}{}^{\prime }\cdot \log\left(\frac{\sigma_{0}+\Delta \sigma }{\sigma_{0}}\right)+\Delta \sigma \cdot b\cdot \left(1-e^{-c\cdot t^{\prime }}\right)+E_{DG}\cdot \left(1-e^{-d\cdot t^{\prime \prime }}\right)$$

where *ε*—deformation, Δ*H*—settlement, *H*—initial height of the landfill, *C_C_*′—compression ratio, *σ*_0_—initial vertical stress, Δ*σ*—change in vertical stress, *b*—coefficient of mechanical creep, *c*, *d*—rate constants for mechanical creep, *E_DG_*—total amount of strain that can occur as a result of biological decomposition, *t*′—time since application of the stress increment, and *t″*—time since placement of waste in the landfill.

The MSW settle during the stage of disposing process, but also after the period of complete exploitation of a landfill. Total displacements on landfills could reach nearly 30–40% of the initial height of a structure [30]. The studies from Estonia and Romania [44] revealed that the degree of the settlements is typically in the range of 5–15% of the landfill height, depending on the content of OM in wastes. They also stated that the major settlements last up to three years. The same phenomena of the majority of settlements occurring in the first few years after landfill operation was confirmed by the recent study performed in Istanbul [13]. For comparison, Esteban-Altabella et al. [29] reported that the initial settlement observed in a landfill in East Spain is about 13.3%, whereas the primary and secondary settlements will reach 9.00% in the first stage, 6.90% in the second stage, and 5.48% in the third stage of the closure phase.

### 2.2. Description of the Study Area—MSW Landfill Štěpánovice

**Figure 1:**
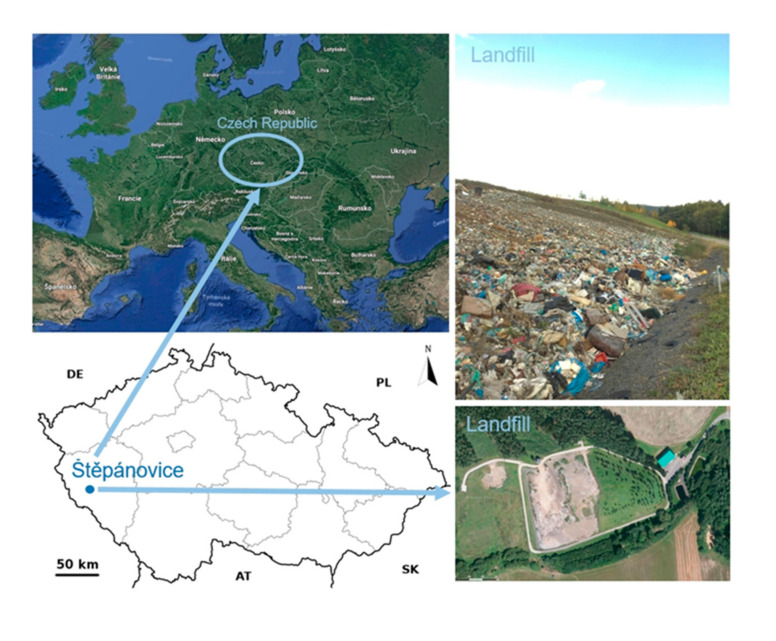
The location of the Štěpánovice landfill.

The Štěpánovice landfill (49.4249858 N, 13.2853425 E) (Figure 1) is operated by the company waste management of the town of Klatovy. It is situated 1000 m north of the Štěpánovice village and 1000 m south of the Dehtín village. The landfill is located in the northern part of a widely open, W-E oriented valley. The watercourse crossing the lower edge of the area is a left-bank tributary of the Točnický Brook, which is a right-bank affluent of the Úhlava River. The upper edge of the landfill premises links up with a forest stand. Lands on the southern part of the landfill are used for agricultural purposes. The landfill is situated on the slope, in the northern direction from the valley axis. Before the landfill construction, the plot was originally managed as a meadow. The area of the landfill premises is situated at an altitude of 421 m above sea level. Pursuant to valid legislation, the facility is designed for waste disposal at the terrain level or beneath code D1. The managed landfill in Štěpánovice is a landfill of the S-OO (other waste) group, subgroup S-OO3, i.e., the category of other waste including waste with a substantial content of organic, biologically degradable materials and wastes. The Štěpánovice landfill is operated in line with the Integrated Permit Decision, issued by the Regional Office of the Pilsen Region on 14 October 2003. Maximum spot height of landfill filling is 443 m a.s.l. Total designed landfill capacity is 275,000 m^3^. The proper landfill body is regularly extended by individual successive stages. This procedure respects the initially declared general project that was subjected to the process of an environmental impact assessment. The oldest part of the landfill body was technically and biologically reclaimed earlier with the reclaimed area totalling 8750 m^2^.

The average annual amount of waste deposited in the Štěpánovice landfill is ca. 20,000 × 10^3^ kg. In recent years, a slightly reduced amount of deposited waste by about 2000 × 10^3^ kg·year^−1^ was recorded, which means that the currently deposited average annual amount of waste is ca. 18,000 × 10^3^ kg. The percentage composition of deposited waste is as follows: 65.8% municipal waste, 19% waste serving for the landfill technical security, and 15.2% waste from the category of “other waste.”

### 2.3. Input Data Analysis

Monitoring of coordinates’ displacement in a landfill can be carried out using traditional geodetic measurements or by GNSS (Global Navigation Satellite Systems) methods. The use of GPS (Global Positioning System) allows for multiple reduction of measurement time and obtaining coordinates for a very large number of benchmarks. The results of measurements can be used both for ongoing operation of the landfill, target shaping of its body, and obtaining waste deformation parameters (“back analysis”) for the purposes of modelling the landfill deformation.

The subject of the study was monitoring of the landfill body stability in the given time period from 2013–2016. The selection of the measurement method was dependent on three basic factors: the speed of changes occurring on the investigated structure, the type of displacements determined, and the required measurement accuracy. The available methods include the “classical” approach based on surveying the network of geodetic benchmarks, the GNSS method, the laser scanning method, or the method of terrestrial radar interferometry. The GNSS method and the method of measuring angular-linear networks allow for periodic measurements and also measurement in real-time by using GNSS receivers and robotic total stations. These systems can work independently of each other or act as a single monitoring system. In the integrated monitoring system, we can also use object deformation sensors such as inclinometers, strain sensors, or fiber optic sensors. This type of monitoring system has been described by Liu et al. [45], which concerns the monitoring of underground structures in the reclamation area. The integrated monitoring system is used not only to determine displacements, but also informs us about the degree of security risk and helps us make early decisions to prevent a construction disaster. In the case of a landfill, a special factor influencing the increase in the risk of deformation are long-term heavy rains and storms, which affect its stability.

The GNSS method [46] consists of determining the position of the receiver based on the coordinates of the satellites (at the time of sending the signal) and the pseudo-distance (from the satellite to the receiver), which is determined from the product of the electromagnetic wave velocity and the difference between the receiver clock time and the GPS time. The pseudo-range is determined from the following formula (Equation (14)).

(14) $$\rho =\sqrt{{\left(x_{sat}-x_{rec}\right)}^{2}+{\left(y_{sat}-y_{rec}\right)}^{2}+{\left(z_{sat}-z_{rec}\right)}^{2}}+c*\delta t$$

where *x_sat_*, *y_sat_*, *z_sat_*—satellites coordinates, *x_rec_*, *y_rec_*, *z_rec_*—receiver coordinates, *δt*—error in synchronizing the receiver clock time with the GPS time, *c*—constant velocity of an electromagnetic wave in a vacuum.

**Figure 2:**
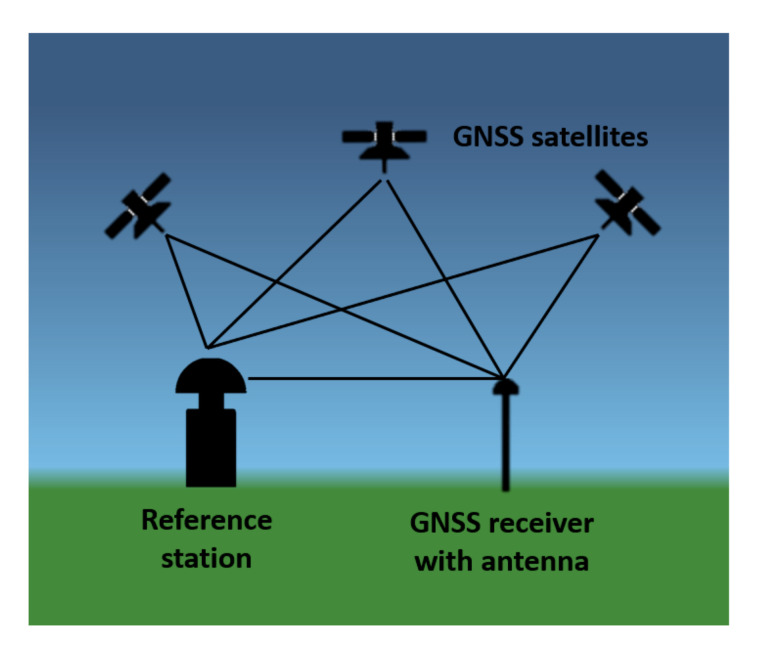
Scheme of Global Navigation Satellite Systems (GNSS) measurement using the differential method.

The receiver position determined in this way is characterized by a low accuracy of a few meters. In order to achieve better results of accuracy in the position of the receiver, differential measurements are used. They consist in precise determination of the relative position (Figure 2). In these measurements, the increments of coordinates between the receiver and the reference station are determined.

The method of measuring geodetic networks, depending on the type of displacements determined, is based on measuring distances and horizontal angles between individual points—horizontal displacements, and measuring elevations between points in the case of vertical displacements. The latter can be determined by trigonometric or geometric levelling. The measurement of the angle-linear network can be supplemented by the GNSS method [47], which allows for determining the spatial position of the measured points in relation to the base station.

The method of terrestrial laser scanning consists of measuring the vertical and horizontal angle and the distance between the measuring station and the measured point. This distance is determined based on the time difference between the output and returning signal and the value of the electromagnetic wave speed. The basic product of laser scanning is a point cloud consisting of coordinates (XYZ) and an intensity value of the reflection parameter. The displacements are appointed by comparing a minimum of two point clouds implemented in the same reference frame.

The ground-based radar interferometry method is one of the latest alternatives to measure object displacements. It is based on a comparison of the phases of the electromagnetic wave reflected from an object, registered at different times from the same measuring position. Based on the phase shift determined in this way, the displacement is calculated according to the formula below (Equation (15)).

(15) $$d=-\frac{\lambda }{4\pi }\Delta \varphi$$

where *d*—displacement, *λ*—electromagnetic wavelength, Δφ—phase shift.

The accuracy of this method depends on the intensity of the reflected signal, which decreases as the distance to the tested object increases. As a result of the measurements, we obtain a map of the difference of signal reflection intensity for the entire object surface representing the displacements.

To determine the displacements of the landfill due to the highest accuracy of the data obtained, the hybrid network measurement method was used. It is a combination of the “classical” method and the GNSS method. The application of such a solution was determined by the lack of direct visibility between the points of the network. In the GNSS measurements, the static method was used due to the high accuracy of determining the coordinates of the receiver. This method is used in setting up and controlling geodetic networks and in geodynamic research. It allows us to achieve one-millimetre positioning accuracy [48].

**Figure 3:**
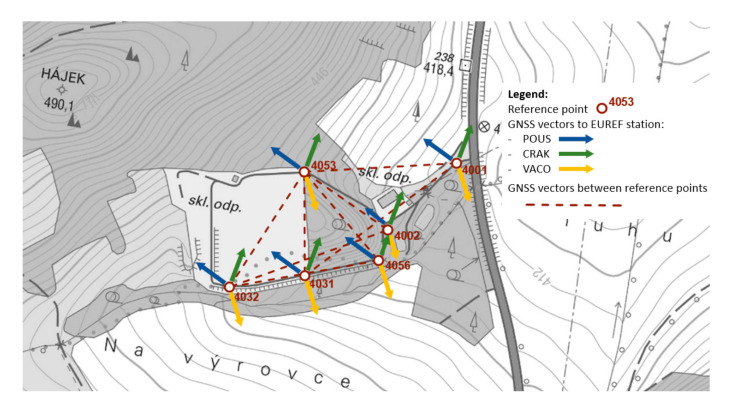
Map of designed control points distribution.

The GNSS static method does not require mutual visibility at measurement points but requires measurement to the same satellites at the same time. Therefore, at least two GNSS receivers should be used for measurements. The development of measurement results requires post-processing, which results in obtaining spatial vectors between points. Measurements using the GNSS method were related to the three closest reference stations of the EUREF network (Regional Reference Frame Sub-Commission for Europe): POUS, CRAK, and VACO. Measurements were made with three GNSS receivers. The network of 3D vectors was supplemented with angular-linear measurements made with a total station in relation to the points determined by the GNSS method. The alignment of the 3D network allowed us to determine the position of the reference points with a situational error not exceeding 2 mm and a height error not exceeding 3 mm. The coordinates of the points were determined in the S-JTSK coordinate system (Unified Trigonometric Cadastral Network) and the Bpv (Baltic Sea vertical datum) altitude system in the Czech Republic. Figure 3 shows the mutual distribution of the designed network points and the measured vectors between these points as well as the vectors to the reference stations of the EUREF network.

**Figure 4:**
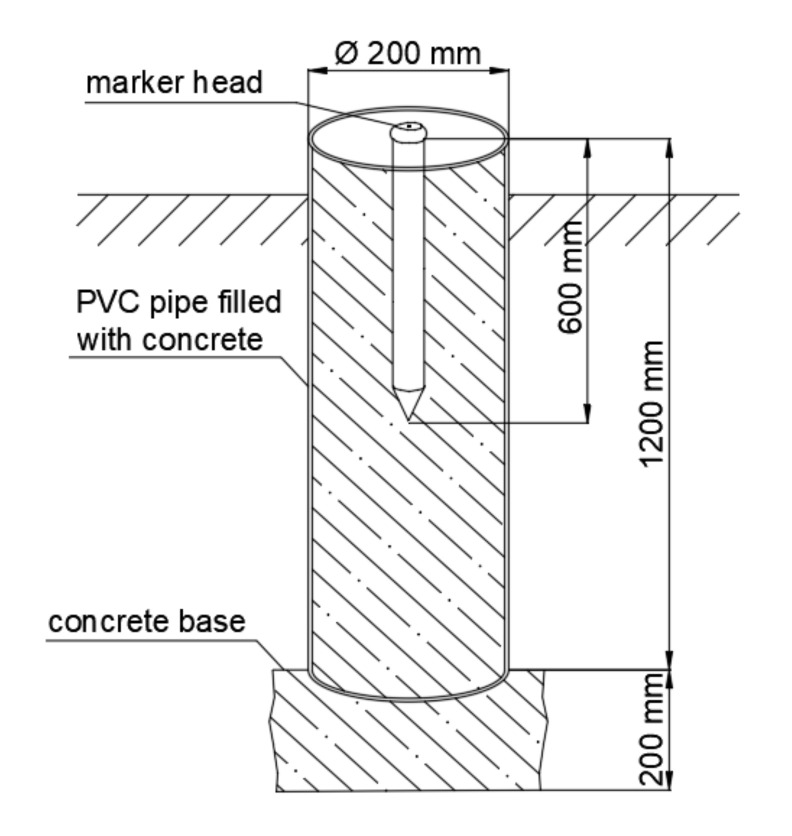
Scheme of control network points stabilisation.

The stabilization of the geodetic control network points required ensuring the stability of their foundation. For this reason, these points were located outside the impact zone of the object (landfill) in places with a stable substrate, while taking into account the geometric conditions that the network of points should meet. Concrete poles 140 cm long and 20 cm in diameter were used to stabilize the points with a metal pin ended with a head, as shown in Figure 4. Six reference points were stabilized in the landfill area.

**Figure 5:**
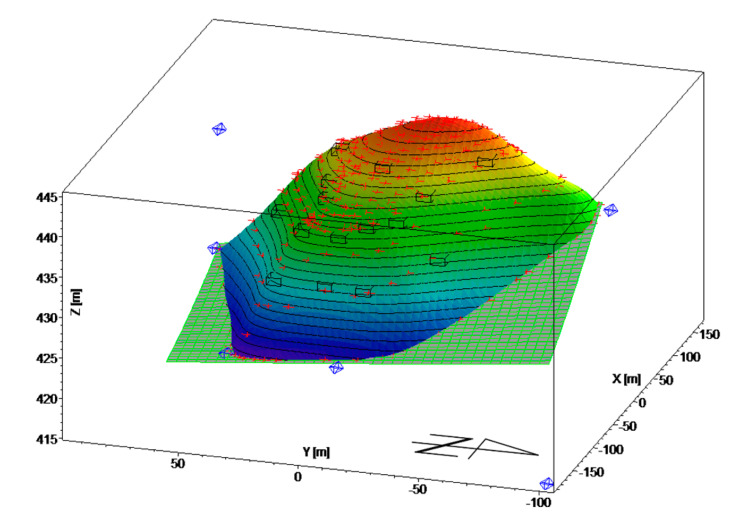
3D location of fundamental points, shape of landscape body, and subsoil.

**Figure 6:**
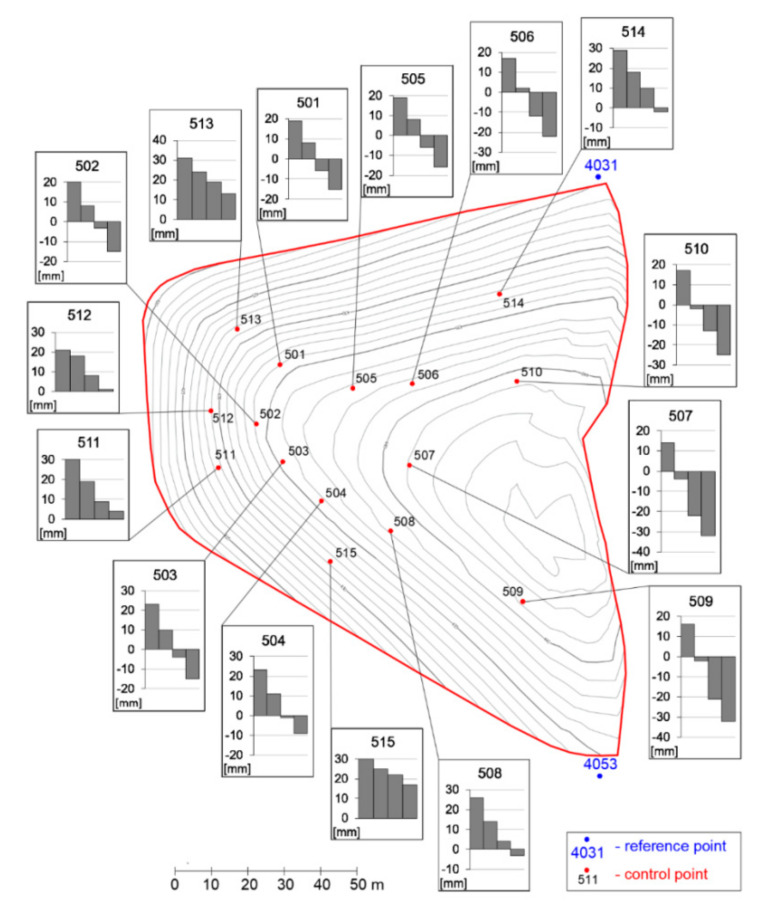
Map of vertical displacements of the landfill surface.

**Figure 7:**
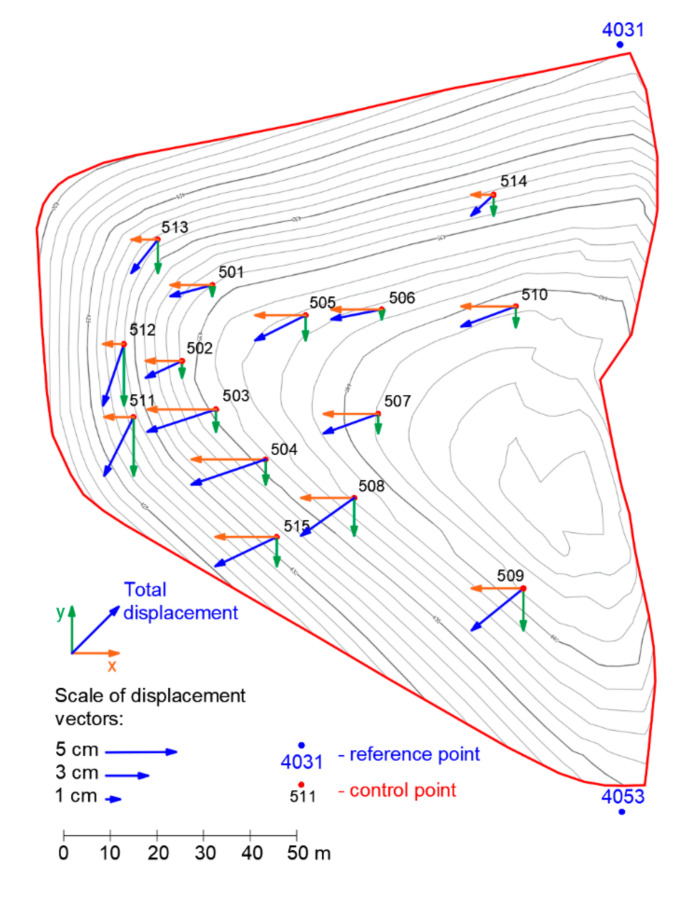
Map of horizontal displacements of the control points on the landfill surface.

In order to monitor the landfill, 15 monitoring points were defined on the landfill body surface (symbol—black cubes on Figure 5), whose position have been precisely recorded for the period of four years (the measurements were carried out in five measuring cycles at annual intervals). In addition to the landfill body, the location of six points was aligned, which served as reference points (symbol—blue octahedrons, see Figure 5). These points served as fixed locations for aligning points defining the landfill body surface and, particularly, for the determination of the displacement of moving points. The landfill body was defined by means of 261 points (symbol—red crosses). All calculations necessary to determine the shape of the landfill body, the profile of the subsoil, and the mathematical model of the movement of the landfill body were performed in Maple 2019. The software was also used to create the following graphs. A 3D image of the location of the defined fundamental points, shape of the landfill body, and ground surface is presented in Figure 5. Vertical displacements of monitored points on the landfill surface are shown in Figure 6, while horizontal movements are shown in Figure 7.

## 3. Results of Displacement Modelling and Discussion

To define the fundamental points, a mathematical model of the landfill body displacement has been developed. Individual coordinates of the landfill body points were used for the calculation of 46 regression function coefficients (Equation (16)).

(16) $$z\left(x,y\right)=\;e^{-P8\left(x,y\right)}+C_{0}$$

where *P*8(*x*,*y*) is a general polynomial of the eights degree in *x*,*y* variables shaped as in Equation (17).

(17) $$P_{8}\left(x,y\right)=C_{45}x^{8}+C_{44}y^{8}+C_{44}x^{7}y+C_{43}x^{6}y^{2}+\cdots C_{38}x^{7}+C_{37}x^{6}y+\cdots C_{43}x+C_{44}y+C_{45}$$

This equation approximates the height of landfill body surface with a high accuracy. *C*_0_*–C*_45_ coefficients were calculated by using the Gauss-Newton iteration method [49,50]. Subsequently, a mathematical model of the shape of the landfill body subsoil was created in which 52 points on the landfill body perimeter and six fixed points were used to calculate regression function coefficients shaped as in Equation (16), while very precisely modelling the subsoil height (Equation (18)).

(18) $$Z\left(x,y\right)=425.6128671+0.0001060146934x^{2}+0.00001136185677y^{2}+0.07476964210\;x-0.04993607926\;y-0.0002601374324xy$$

**Figure 8:**
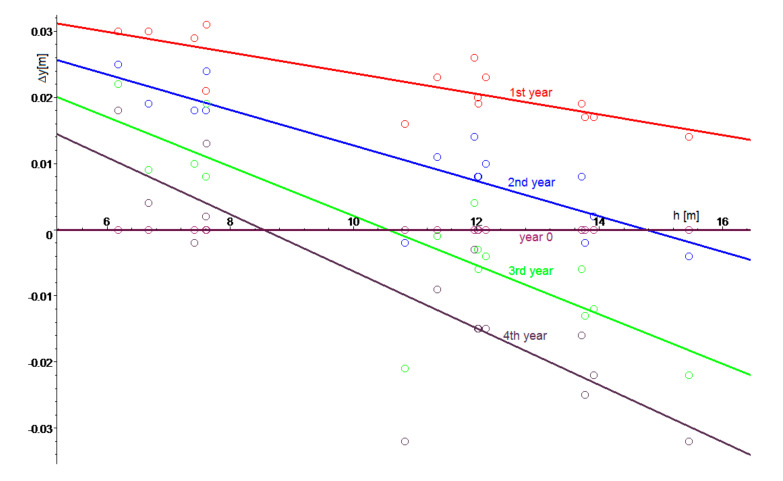
Vertical displacement in dependence on the material layer thickness and time.

In order to establish the subsoil height, vertical displacement of the landfill body was studied where the thickness of the layer of landfilled waste (*h*) can be determined for any point on the landfill surface from the difference of functions (1)—denoting the landfill body height, and (2)—defining the subsoil height. Displacement is divided into a horizontal and vertical direction. Vertical displacement will be modelled by the function depending on the time and thickness of the waste layer. Measured displacements of fixed points were distributed to a horizontal displacement in the direction of the *x*,*y* plane and to vertical displacements in the direction of the *z* axis. Figure 8 shows a diagram plotted for the respective years, which shows the vertical displacement of individual points (Δ*y*) in dependence on the layer thickness of stored waste (*h*).

The diagram indicates a relationship between the vertical displacements, waste layer thickness, and time, which can be mathematically described by means of the regression function (Equation (19)).

(19) $$\Delta y\left(h,t\right)=0.005455826410\;t\;h-0.0003368687824\;t\;h^{2}-0.001227089868\;t^{2}h+0.00006562182244\;t^{2}h^{2}$$

**Figure 9:**
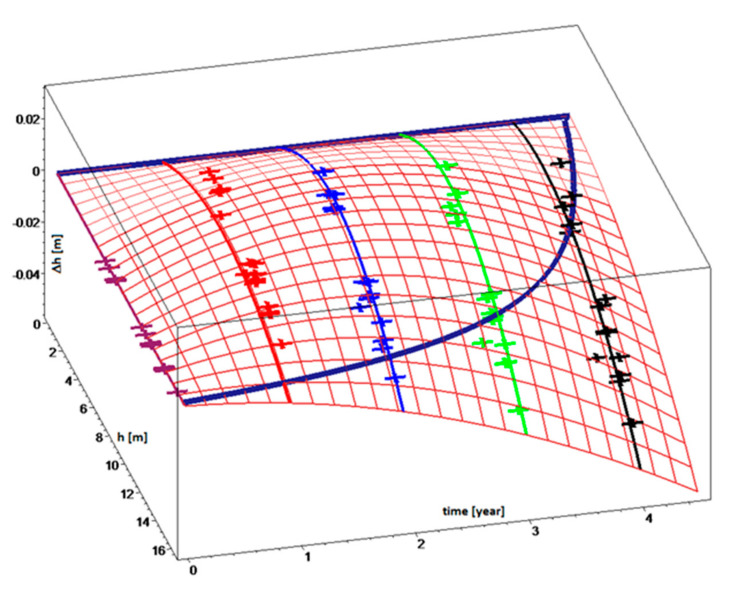
Vertical displacement in dependence on waste layer thickness and time.

The mathematical shape of the function (Equation (19)) shown in Figure 9 indicates that the function (Equation (19)) effectively characterizes the landfill behaviour with the coefficient of reliability reaching 56%. The waste deposited in the landfill is subjected to vertical displacements, which depend on the waste layer thickness and time. Regression analysis describing the shape of the landfill is based on data obtained by triangulation measurements and monitoring basis. The result is a function *z*(*x*,*y*), which, in this case, describes the shape of the slope’s surface. Therefore, any factors influencing surface deformation cannot be represented in this model. This is to present a different approach on the modelling in a case of a lack of access to much more sophisticated data like elasticity parameters of waste material, and to evaluate how reliable such an estimation would be.

**Figure 10:**
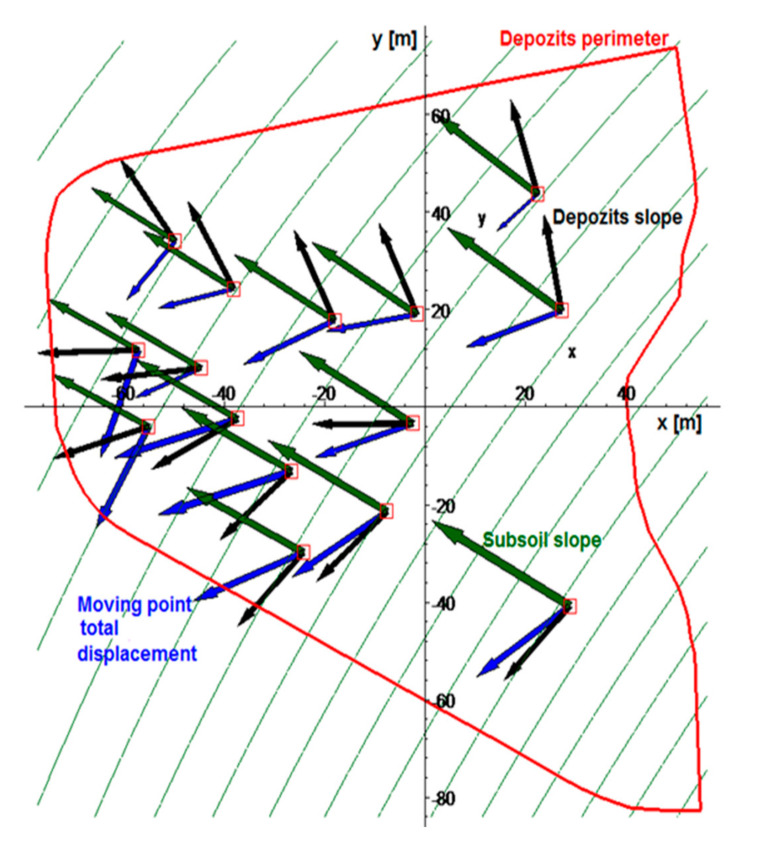
Measuring point displacement directions in reference to inclination of the landfill and deposit slopes.

Apart from the vertical displacement of the waste, the horizontal displacements could be compared with the directions of the landfill surface gradient and gradient of the ground level (Figure 10).

Figure 10 shows that total displacement is not parallel to the vectors of the landfill surface gradient—the landfill slope did not expose any failure signs. The displacement is not parallel to the vectors of subsoil gradient either. The landfill does not move. When viewed from above, only the horizontal shift is visible. This displacement is not parallel to the landfill slope. Vectors are heading the horizontal displacement of the landfill body and are not caused by any type of a landslide. Moreover, the size of the horizontal displacement is rather negligible when compared to the dimensions of the landfill body. Nevertheless, coefficients of the regression function, which explains the course of the vector field of the total displacement of moving points, could be calculated. If a regression function is selected, characterizing the vector fields of resulting displacement in the form of the equation below (Equation (20)).

(20) $$V\left(x,y\right)=\left[\begin{array}{l} Cx_{1}+x^{3}Cx_{2}+y^{3}Cx_{3}+x\;Cx_{4}+y\;Cx_{5}\;+x^{2}Cx_{6}+y^{2}Cx^{7}+x\;y\;Cx_{8}+x\;y^{2}Cx_{9}+x^{2}y\;Cx_{10}\\ Cy_{1}+x^{3}Cy_{2}+y^{3}Cy_{3}+x\;Cy_{4}+y\;Cy_{5}+x^{2}Cy_{6}+y^{2}Cy_{7}+x\;y\;Cy_{8}+x\;y^{2}Cy_{9}+x^{2}y\;Cy_{10} \end{array}\right]$$

**Figure 11:**
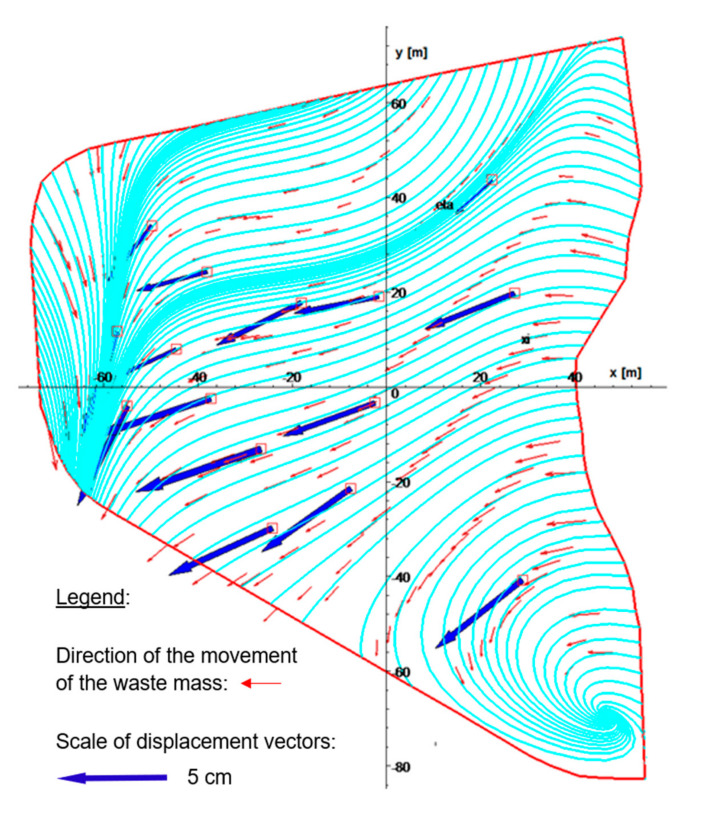
Trajectories of landfill material displacement.

The method of least squares can be used to calculate coefficients *Cx*_1_…*Cx*_10_ and *Cy*_1_*…Cy*_10_, and then to plot trajectories of the displacement of landfilled waste by means of the Runge-Kutta method [51]. Vectors in Figure 11 show that the curves of waste displacement are in excellent agreement with the measured total displacement, which is illustrated using blue arrows. Practically, all curves corresponding to waste displacement are perpendicular to the active edge of the landfill onto which new waste is delivered. This indicates that the waste displacements are caused by the weight of the newly delivered waste and by pressures emerging during its compaction and transport. The whirl occurring in the right lower part of the landfill (Figure 11) on coordinates (45, 69) results from the non-existence of any adjacent moving point determining the vector field of total displacements.

## 4. Conclusions

Operation and remediation of the landfill undergoes various complex displacement mechanisms controlled by hydraulic, mechanical, chemical, and biological processes. An understanding of coupled interactions in the waste body on the landfill is critical in designing a stable, effective, and operational safe site. Landfill deformations impact the design, operation, closure, development, and safe future use of the entire site [52]. The current practices associated with mathematical modelling as well as long-term monitoring of landfill displacement(s) are mostly empirical and limited to site conditions.

The spatially and temporally varied waste composition as well as heterogeneous and anisotropic nature of landfill body results in atypical differential landfill displacement. In the paper, the numerical modelling of the landfill undergoing coupled processes is presented.

Based on the geodetic survey made on the MSW Landfill in Štěpánovice in 2013–2016 and performed calculations, it is possible to state that the landfill is expanding if the waste layer thickness is not too thick (the landfill body expands slightly vertically). Specifically, if the waste layer thickness is less than 16.2 m, the waste expands at the beginning after deposition. The size of the landfill expansion and its duration depend on the waste layer thickness. On the other hand, if the waste layer thickness is more than 16.2 m, waste expansion will never occur. In the course of time, the waste gets further compacted by its own weight. The waste expansion takes place for the time of a maximum Of 4.5 years. After the lapse of this time, the waste would settle within the complete landfill volume. Maximum expansion of 2 cm in size occurs for the waste layer of 7.3 m in thickness and within 2 years after the waste deposition on the landfill. This shows that, after some time, the waste expansion would stop, and the waste would be further compacted by its own weight. The calculation indicates that the horizontal waste displacement is caused by the pressure of the newly delivered and compacted waste. The landfill does not move as a whole, neither its embankments slip down even at places with the greatest gradient of landfill embankment or subsoil. The landfill is stable because the total horizontal displacement in both a horizontal and vertical direction is negligible as compared to the dimensions and height of the landfill.
